# Supplementary material for: Trioxane-based MS-cleavable cross-linking mass spectrometry for profiling multimeric interactions of cellular networks
Source: Nat Commun. 2025 Jul 1;16:5585. doi: 10.1038/s41467-025-60642-3 (PMC12215968; doi:10.1038/s41467-025-60642-3)
Supplement: Supplementary file 1 — Supplementary Information [file 41467_2025_60642_MOESM1_ESM.pdf]

SUPPLEMENTARY INFORMATION

Trioxane-based MS-cleavable Cross-linking Mass Spectrometry for Profiling Multimeric Interactions of Cellular Networks

Clinton Yu[1], Eric Novitsky[2], Sree Ganesh Balasubramani[3], Xiaorong Wang[1], Xiyu Shen[1], Qin Yang[1,4], Scott Rychnovsky[2], Ignacia Echeverria[3,5], Lan Huang[1]*

[1]Department of Physiology & Biophysics, University of California, Irvine, Irvine, CA 92697, USA

[2]Department of Chemistry, University of California, Irvine, Irvine, CA 92697, USA

[3]Department of Cellular and Molecular Pharmacology, University of California, San Francisco, San Francisco, CA 94158, USA.

[4]Department of Medicine, University of California, Irvine, Irvine, CA 92697, USA

[5]Quantitative Biosciences Institute, University of California, San Francisco, San Francisco, CA 94158, USA

*Correspondence should be addressed to Dr. Lan Huang (lanhuang@uci.edu)

Medical Science I, D233

Department of Physiology & Biophysics

University of California, Irvine

Irvine, CA 92697-4560

Phone: (949) 824-8548

Fax: (949) 824-8540

TABLE OF CONTENTS

Supplementary Figure 1 3

Supplementary Figure 2 4

Supplementary Figure 3 5

Supplementary Figure 4 6

Supplementary Figure 5 7

Supplementary Figure 6 8

Supplementary Figure 7 9

Supplementary Figure 8 10

Supplementary Figure 9 11

Supplementary Figure 10 12

Supplementary Figure 11 13

Supplementary Figure 12 14

Supplementary Figure 13 15

Supplementary Data 16

References 17

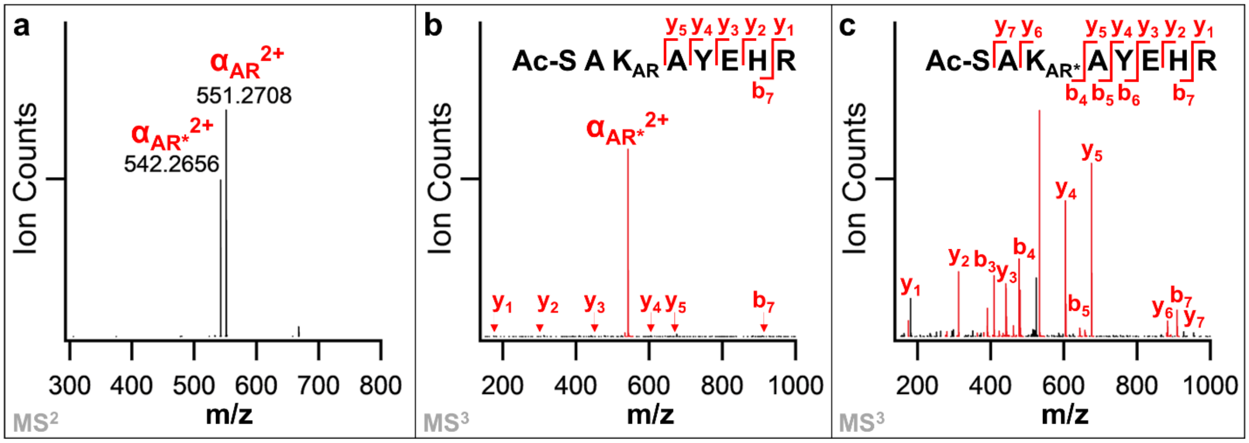

Supplementary Figure 1. MS^n analyses of TSTO dead-end modified Ac-SR8. (a) MS^2 analysis of the dead-end modified Ac-SR8 [α_{DN}] (*m/z* 667.3163^{2+}) yielded a dominant ion pair: α_{AR}/α_{AR*}, which has a mass difference of 18.02 Da. As the aldehyde moiety (AR) is prone to water loss, the dehydrated aldehyde moiety (AR*) was detected. **(b)** MS^3 analysis of α_{AR} (*m/z* 551.2708^{2+}) identified its sequence as Ac-SAK$_{AR}$AYEHR, in which K was modified by the aldehyde moiety (AR). **(c)** MS^3 analysis of α_{AR*} (*m/z* 542.2656^{2+}) identified its sequence as Ac-SAK$_{AR*}$AYEHR, in which K was modified by the dehydrated aldehyde moiety (AR*). Source data are provided as a Source Data file.

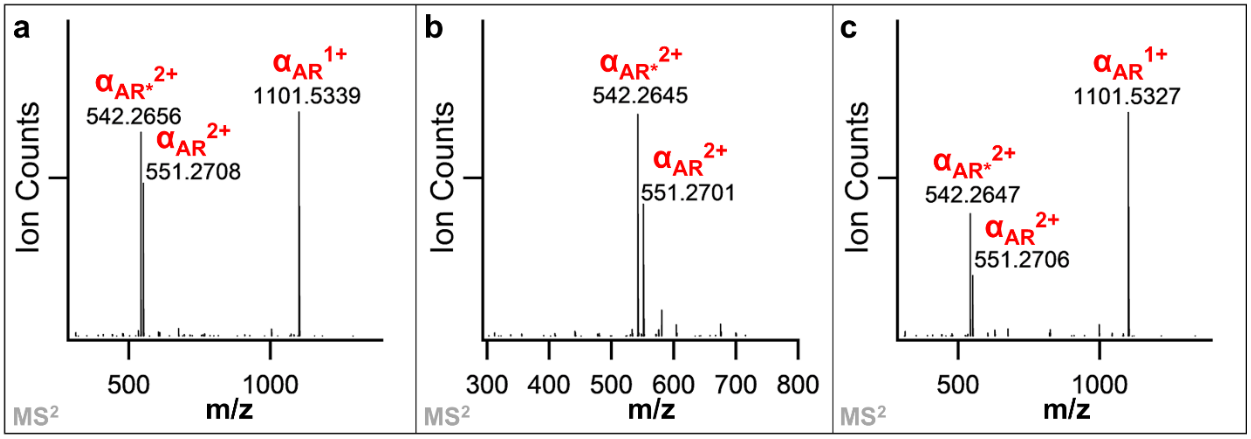

Supplementary Figure 2. MS^2 fragmentation characteristics of TSTO inter-linked Ac-SR8. **(a-b)** MS^2 spectra of inter-linked Ac-SR8 homodimers [α-α]: **(a)** triply (*m/z* 773.7071^{3+}) and **(b)** quadruply charged (*m/z* 580.5319^{4+}). **(c)** MS^2 spectrum of the inter-linked Ac-SR8 homotrimer [α,α,α] (*m/z* 826.6508^{4+}). Note: AR: aldehyde moiety; AR*: dehydrated aldehyde moiety. Source data are provided as a Source Data file.

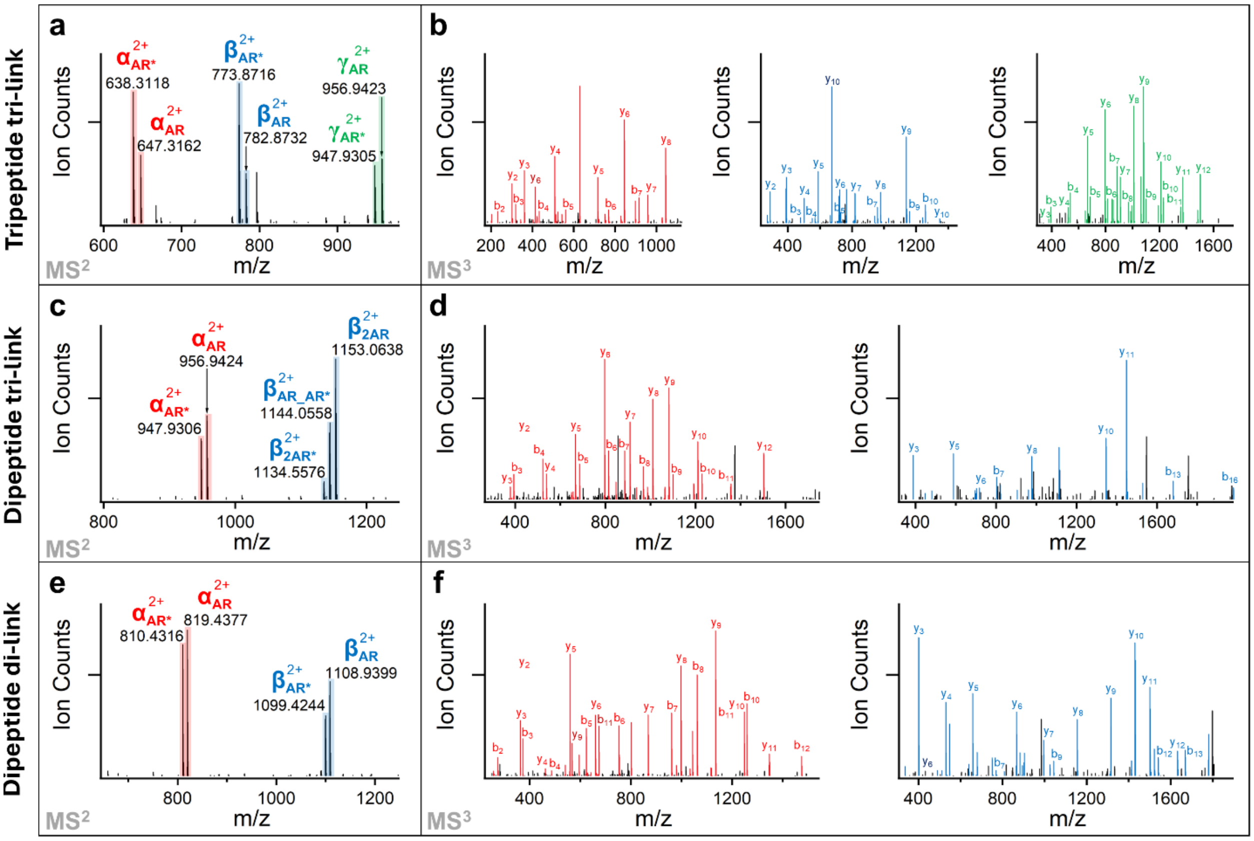

Supplementary Figure 3. MS^n Analyses of representative TSTO inter-linked peptides of BSA. (a) MS^2 spectrum of a tripeptide tri-link [α, β, γ] (*m/z* 795.7120^{6+}) in which a series of dominant ions corresponding to α_{AR}, β_{AR}, and γ_{AR} fragments were detected. **(b)** MS^3 sequencing of α_{AR}* (*m/z* 638.3130^{2+}), β_{AR}* (*m/z* 773.874^{3+}), and γ_{AR}* (*m/z* 947.934^{7+}) enabled their identification as CASIQK$_{AR*}$FGER, VTK$_{AR*}$CCTESLVNR, and LAK$_{AR*}$EYEATLEECCAK, respectively, signifying a tripeptide TSTO tri-link among BSA lysines K228, K374, and K498. **(c)** MS^2 spectrum of a dipeptide tri-link [α-β_2] (*m/z* 1054.7530^{4+}), in which two sets of dominant ion species were observed: α_{AR}/α_{AR*}, and β_{2AR}/β_{AR_AR*}/β_{2AR*}. **(d)** MS^3 analyses of α_{AR*} and β_{2AR*} identified their sequences as LAK$_{AR*}$EYEATLEECCAK and TPVSEK$_{AR*}$VTK$_{AR*}$CCTESLVNR, signifying a dipeptide tri-link [BSA:K374 - BSA:K495, K498]. **(e)** MS^2 spectrum of a dipeptide bi-link [α-β] (*m/z* 992.7017^{4+}), in which two dominant ion pairs were detected: α_{AR}/α_{AR*}, and β_{AR}/β_{AR*}. **(f)** MS^3 analyses of α_{AR*} (810.4316^{2+}) and β_{AR*} (1099.4244^{2+}) determined their sequences as LCVHEK$_{AR*}$TPVSEK and ETYGDMADCCEK$_{AR*}$QEPER, identifying a bi-link between BSA:K117 and BSA:K489. Source data are provided as a Source Data file.

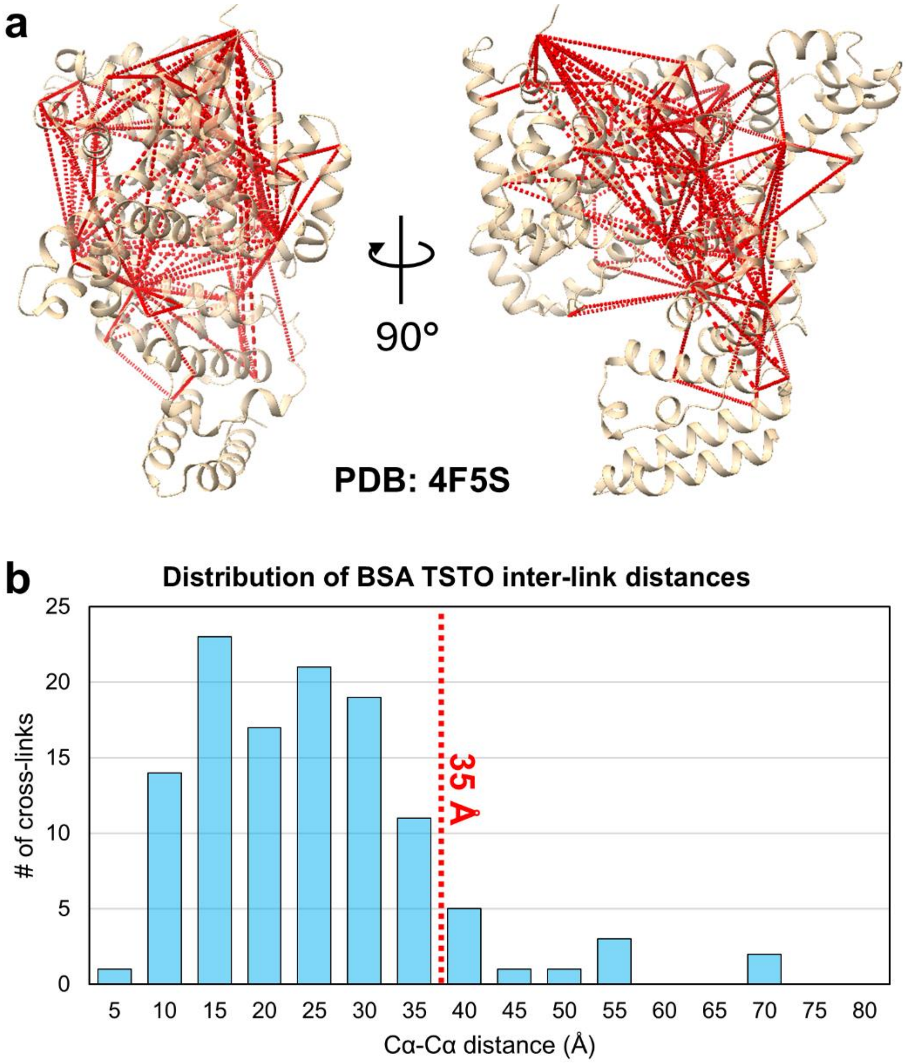

Supplementary Figure 4. 3-D distance mapping of TSTO cross-links of BSA. (a) 118 TSTO cross-links were mapped to a high-resolution structure of BSA (PDB: 4F5S). **(b)** Distance distribution plot of TSTO cross-links, 90% of which were satisfied with Cα-Cα distances below the threshold of 35 Å. Source data are provided as a Source Data file.

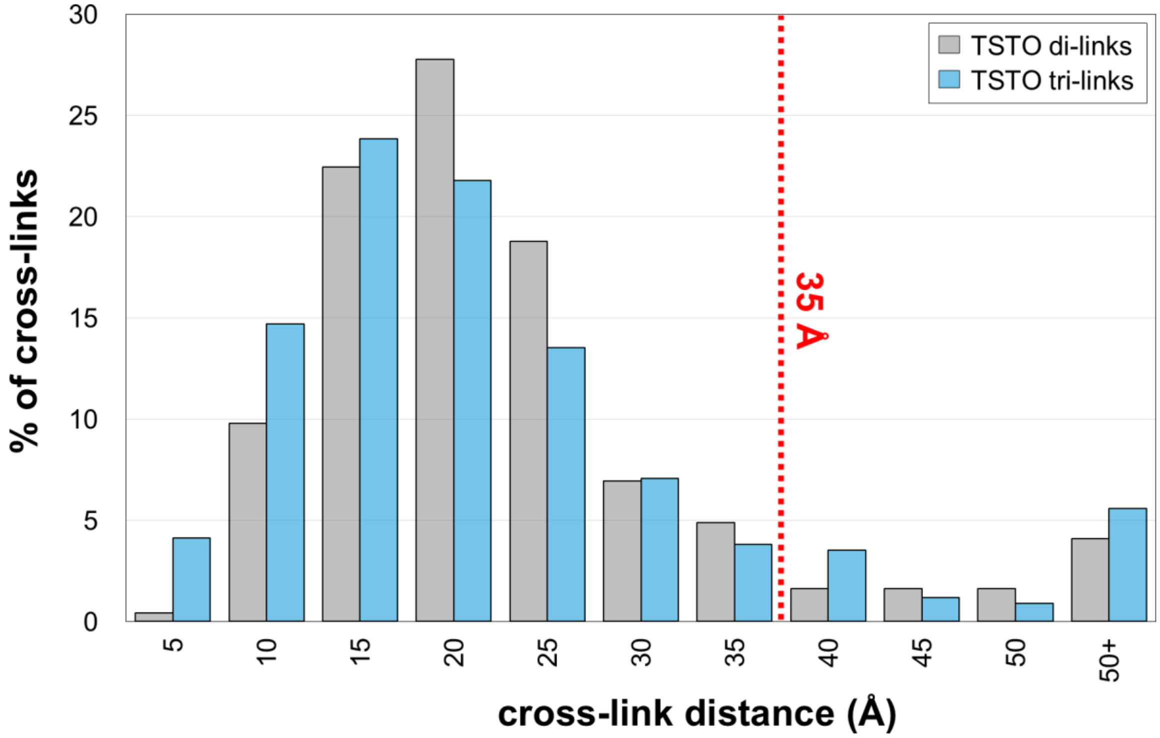

Supplementary Figure 5. Distance distribution plots of unique residue pairs derived from TSTO cross-linking of 26S proteasomes. Unique residue pairs from TSTO bi-links are shown in gray, while those from TSTO tri-links are shown in light blue. Cross-linked residues were mapped onto a high-resolution structure of the 26S proteasome (PDB: 7QY7). Source data are provided as a Source Data file.

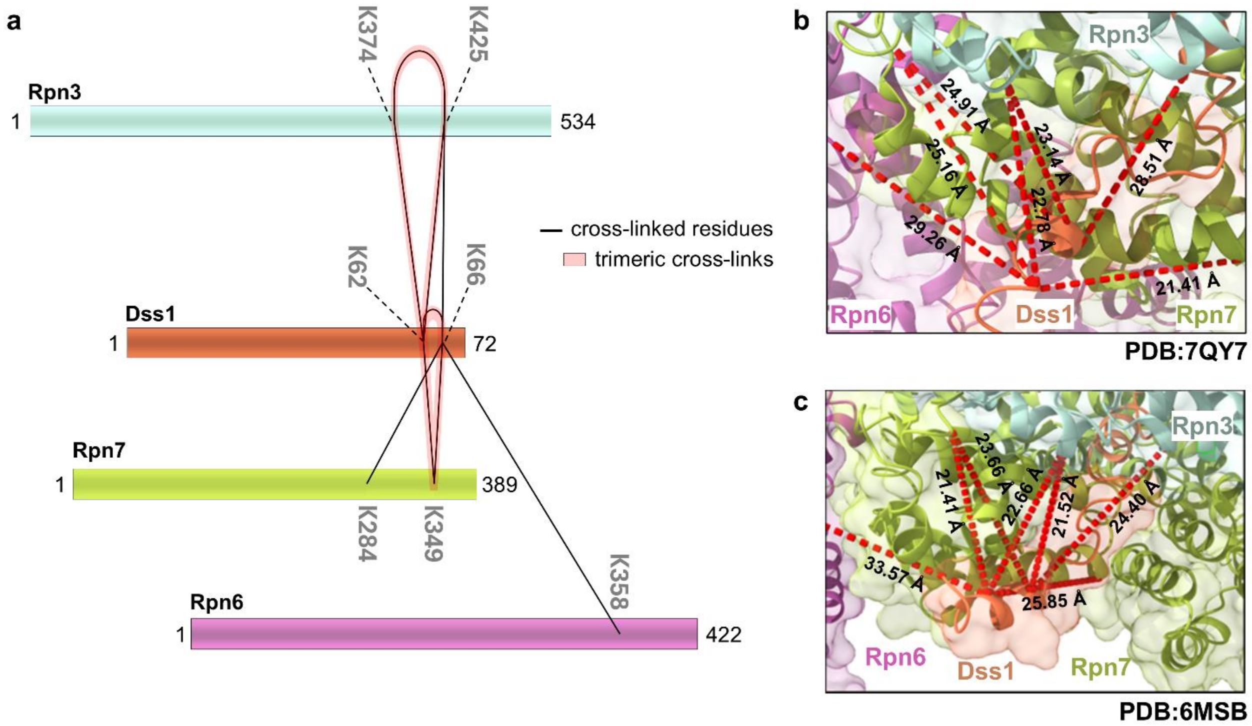

Supplementary Figure 6. Dss1 interactions captured by TSTO cross-linking. (a) 2-D XL-map of Dss1 cross-links to 19S lid subunit Rpn3, Rpn6, and Rpn7. Trimeric interactions are highlighted in red. Dss1-Rpn3, Dss1-Rpn6, and Dss1-Rpn7 cross-links were mapped to the high-resolution 26S proteasome structures: **(b)** PDB:7QY7 [1] and **(c)** PDB:6MSB [2].

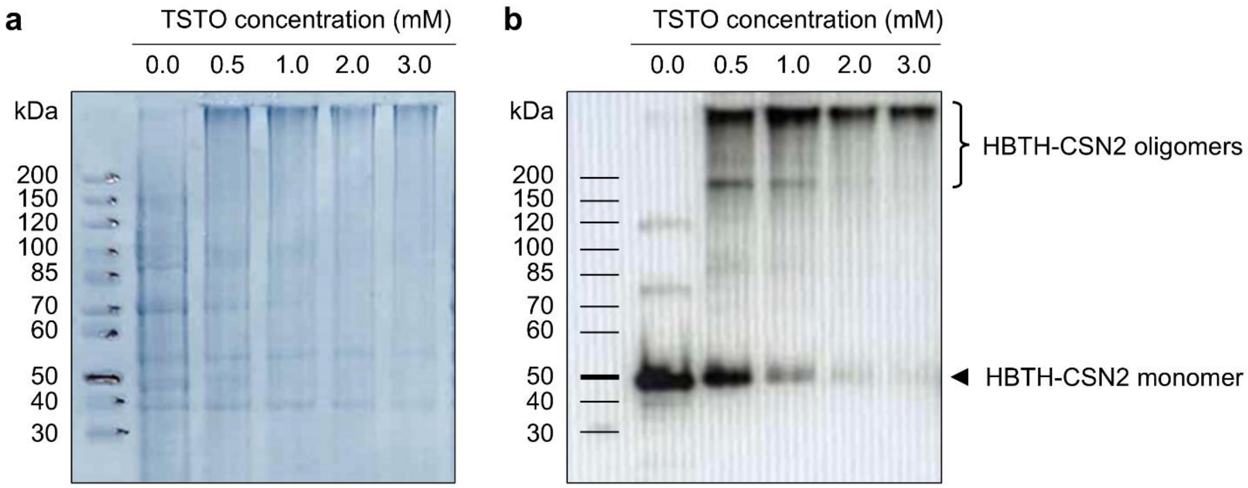

Supplementary Figure 7. Evaluation and optimization of TSTO *in vivo* cross-linking. TSTO cross-linking was tested at various concentrations (0.5-3 mM) using HEK 293$^{\mathbf{HBTH\text{-}CSN2}}$ cells. The cross-linked products were separated by SDS-PAGE, transferred onto a PVDF membrane, and evaluated by **(a)** amido black staining and **(b)** western blot analysis using StrepHRP to probe HBTH-tagged CSN2. The bands corresponding to the oligomers and monomer of HBTH-tagged CSN2 were indicated.

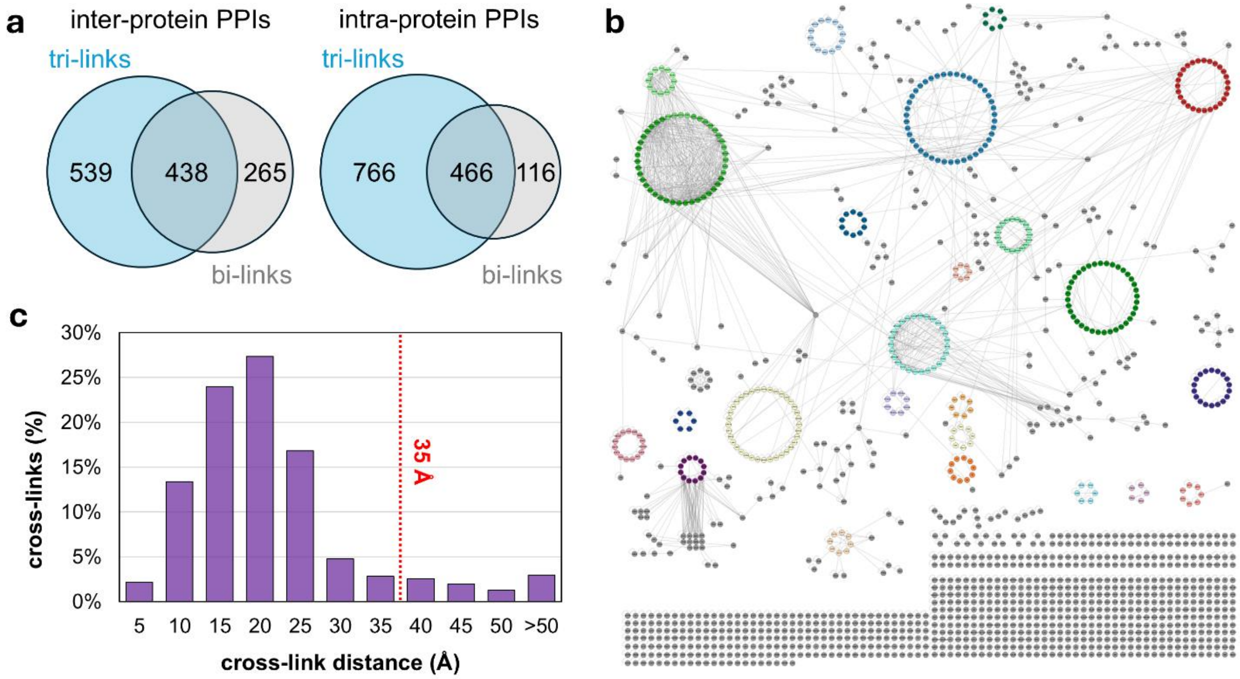

Supplementary Figure 8. 26S TSTO cross-linking analysis. (a) Venn diagrams depicting the overlap of inter- and intra-protein PPIs described by TSTO bi- and tri-links captured from *in vivo* cross-linking. **(b)** Histogram of mapped Ca-Ca distances for 1790 URPs across 539 CORUM complexes; 95% were found to be ≤ 35 Å. **(c)** *In vivo* XL-PPI network of HEK 293 cells derived from TSTO cross-links comprising 1512 nodes connected by 1242 edges. Source data are provided as a Source Data file.

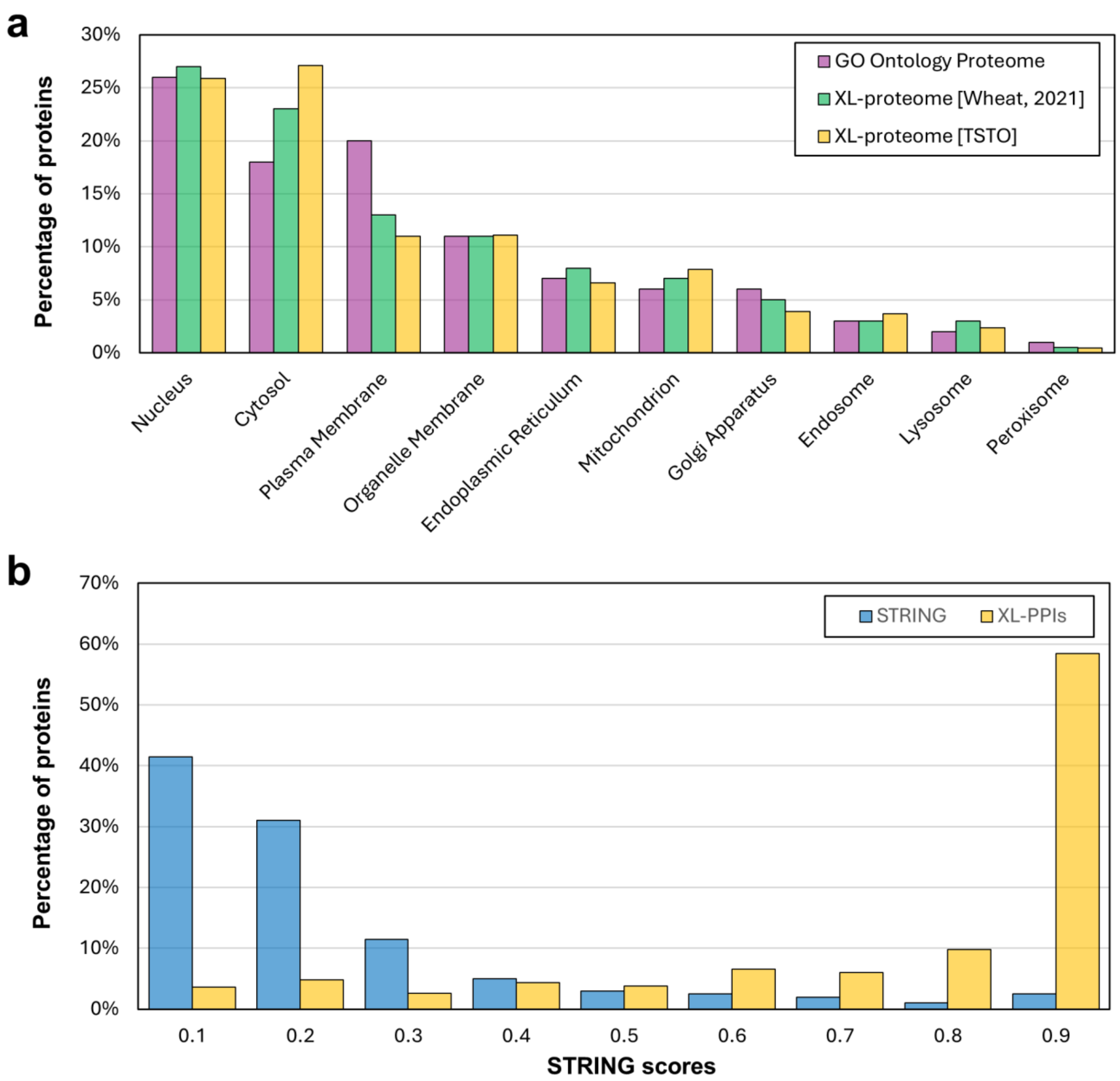

Supplementary Figure 9. Evaluation of the TSTO XL-Proteome. (a) Gene Ontology (GO)[3,4] analysis showing cell compartment distribution of TSTO XL-proteome compared to GO proteome and DSBSO XL-proteome [5]. In both cross-linking datasets, cytosolic proteins are enriched, and plasma membrane proteins have decreased representation compared to the Gene Ontology proteome. (**b)** Distribution of STRING scores for XL-PPIs captured by *in vivo* TSTO cross-linking compared to human PPIs curated within the STRING database. Source data are provided as a Source Data file.

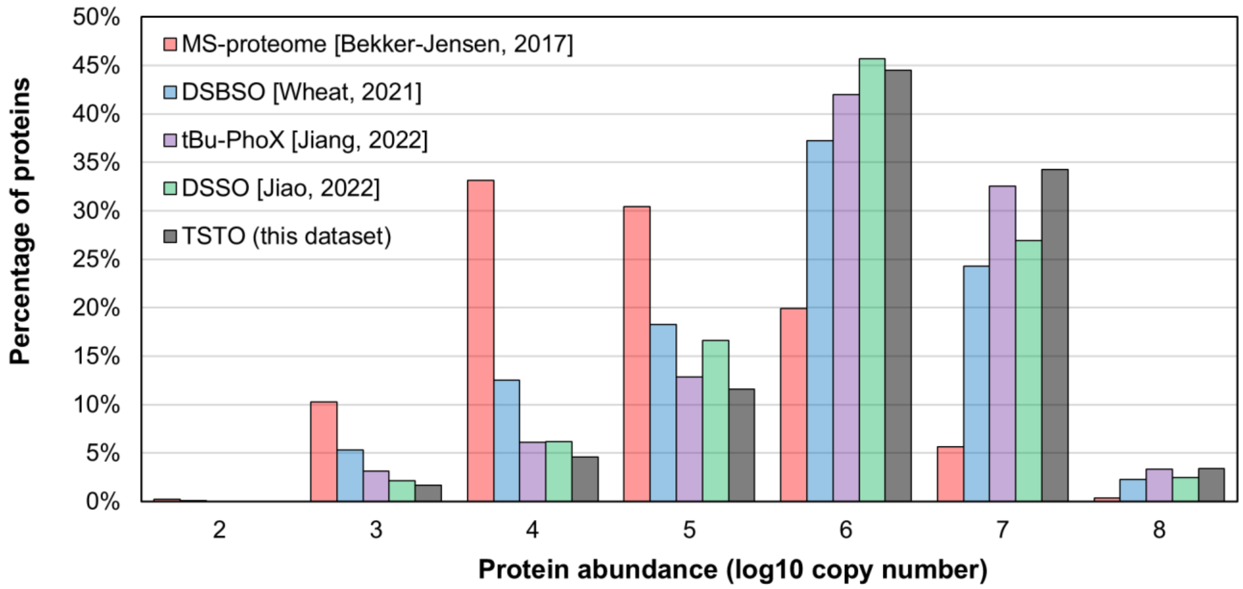

Supplementary Figure 10. Comparison of protein abundances across the TSTO XL-proteome, MS-proteome and several published XL-proteomes. Protein abundance distribution of the human cell MS proteome determined by shotgun proteomics[6] (red) compared to selected XL-proteomes (alkyne-A-DSBSO in vivo dataset from Wheat et al.[5] (blue), tBu-PhoX in vivo dataset from Jiang et al.[7] (purple), DSSO in vitro dataset from Jiao et al.[8] (yellow), and TSTO in vivo dataset from this work (grey)). Source data are provided as a Source Data file.

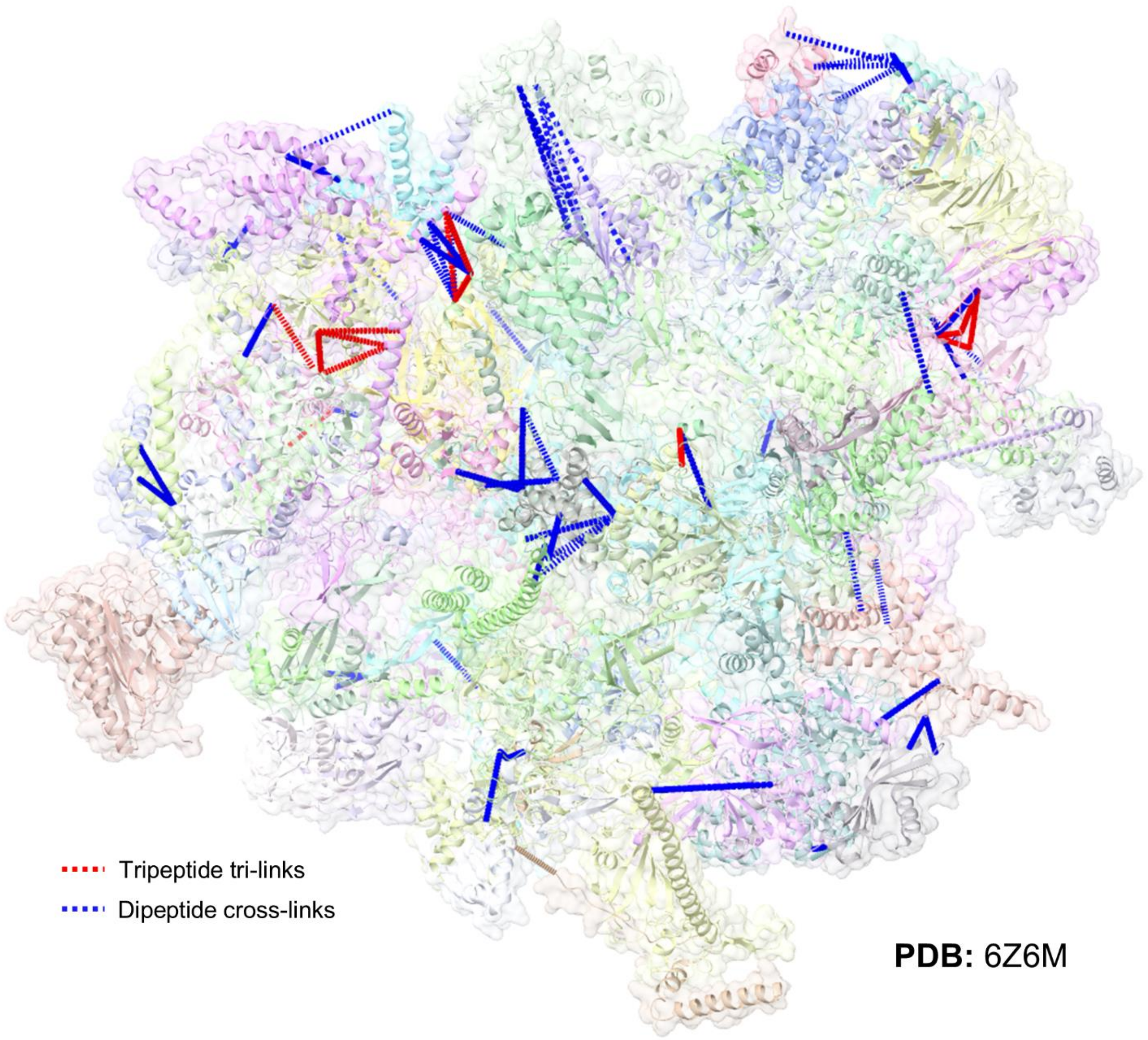

Supplementary Figure 11. Mapping of TSTO cross-links to a high-resolution structure of the 80S ribosomal complex (PDB: 6Z6M[9]). Mapped distances between residues identified in trimeric cross-links are shown in red, while other cross-links are shown in blue. The overall satisfaction rate of mapped cross-links ≤ 35 Å was 96%.

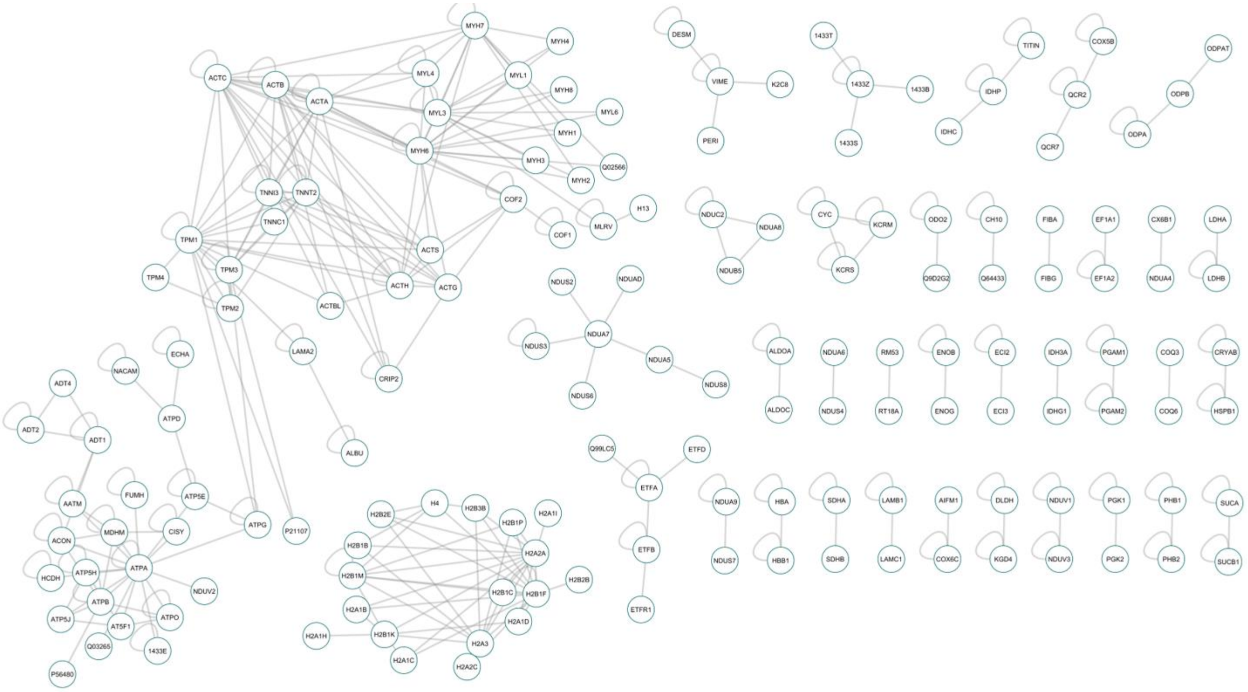

Supplementary Figure 12. TSTO XL-MS data of mouse heart tissue. Interaction network identified by TSTO cross-linking.

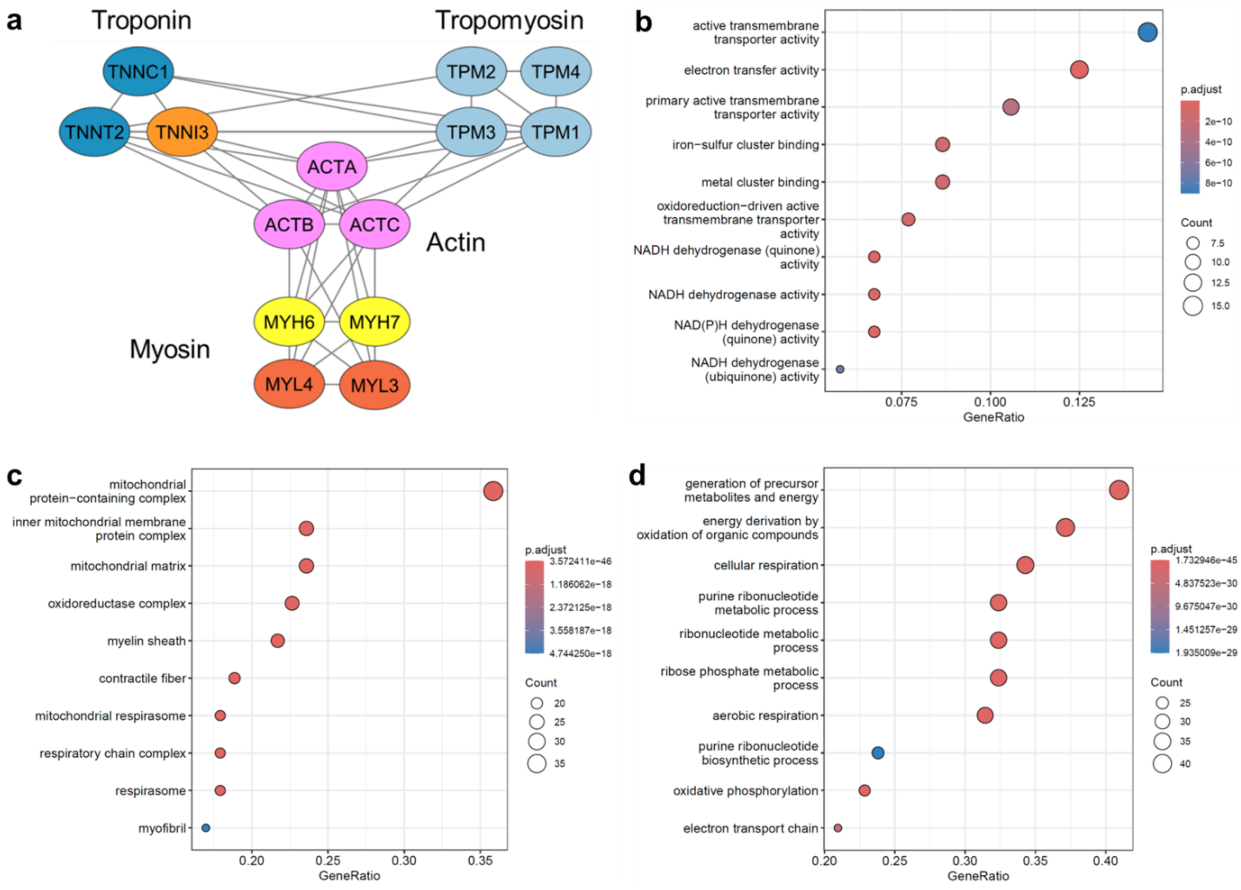

Supplementary Figure 13. Analysis of mouse heart tissue cross-linking. (a) XL-PPI network describing interactions among proteins associated with heart muscle function. **(b-d)** GO enrichment analysis of TSTO XL-proteome describing **(b)** molecular function, **(c)** cellular components, and (d) biological processes associated with heart-specific and enriched proteins. Source data are provided as a Source Data file.

Supplementary Data

Supplementary Data 1. TSTO cross-links identified from cross-linked BSA.

Supplementary Data 2. TSTO cross-links identified from affinity-purified human 26S proteasomes.

Supplementary Data 3. TSTO cross-links identified from in vivo cross-linked HEK 293 cells.

Supplementary Data 4. TSTO cross-links identified from in vivo cross-linked mouse heart tissue.

References

1 Lee, D. *et al.* Molecular mechanism for activation of the 26S proteasome by ZFAND5. *Mol Cell* **83**, 2959-2975 e2957, doi:10.1016/j.molcel.2023.07.023 (2023).

2 Dong, Y. *et al.* Cryo-EM structures and dynamics of substrate-engaged human 26S proteasome. *Nature* **565**, 49-55, doi:10.1038/s41586-018-0736-4 (2019).

3 Ashburner, M. *et al.* Gene ontology: tool for the unification of biology. The Gene Ontology Consortium. *Nat Genet* **25**, 25-29, doi:10.1038/75556 (2000).

4 Gene Ontology, C. *et al.* The Gene Ontology knowledgebase in 2023. *Genetics* **224**, doi:10.1093/genetics/iyad031 (2023).

5 Wheat, A. *et al.* Protein interaction landscapes revealed by advanced in vivo cross-linking-mass spectrometry. *Proc Natl Acad Sci U S A* **118**, doi:10.1073/pnas.2023360118 (2021).

6 Bekker-Jensen, D. B. *et al.* An Optimized Shotgun Strategy for the Rapid Generation of Comprehensive Human Proteomes. *Cell Syst* **4**, 587-599 e584, doi:10.1016/j.cels.2017.05.009 (2017).

7 Jiang, P. L. *et al.* A Membrane-Permeable and Immobilized Metal Affinity Chromatography (IMAC) Enrichable Cross-Linking Reagent to Advance In Vivo Cross-Linking Mass Spectrometry. *Angew Chem Int Ed Engl* **61**, e202113937, doi:10.1002/anie.202113937 (2022).

8 Jiao, F. *et al.* Two-Dimensional Fractionation Method for Proteome-Wide Cross-Linking Mass Spectrometry Analysis. *Anal Chem* **94**, 4236-4242, doi:10.1021/acs.analchem.1c04485 (2022).

9 Wells, J. N. *et al.* Structure and function of yeast Lso2 and human CCDC124 bound to hibernating ribosomes. *PLoS Biol* **18**, e3000780, doi:10.1371/journal.pbio.3000780 (2020).
