# Supplementary material for: Trioxane-based MS-cleavable cross-linking mass spectrometry for profiling multimeric interactions of cellular networks
Source: Nat Commun. 2025 Jul 1;16:5585. doi: 10.1038/s41467-025-60642-3 (PMC12215968; doi:10.1038/s41467-025-60642-3)
Supplement: Supplementary file 2 — Description of Additional Supplementary Files [file 41467_2025_60642_MOESM2_ESM.pdf]

Description of Additional Supplementary Files

File Name: Supplementary Data 1
Description: TSTO cross-links identified from cross-linked BSA.

File Name: Supplementary Data 2
Description: TSTO cross-links identified from affinity-purified human 26S proteasomes.

File Name: Supplementary Data 3
Description: TSTO cross-links identified from in vivo cross-linked HEK 293 cells.

File Name: Supplementary Data 4
Description: TSTO cross-links identified from in vivo cross-linked mouse heart tissue.
