# Supplementary material for: Trioxane-based MS-cleavable cross-linking mass spectrometry for profiling multimeric interactions of cellular networks
Source: Nat Commun. 2025 Jul 1;16:5585. doi: 10.1038/s41467-025-60642-3 (PMC12215968; doi:10.1038/s41467-025-60642-3)
Supplement: Supplementary file 7 — Reporting Summary [file 41467_2025_60642_MOESM7_ESM.pdf]

nature portfolio

Corresponding author(s): Lan Huang

Last updated by author(s): 5/12/2025

Reporting Summary

Nature Portfolio wishes to improve the reproducibility of the work that we publish. This form provides structure for consistency and transparency in reporting. For further information on Nature Portfolio policies, see our Editorial Policies and the Editorial Policy Checklist.

Statistics

For all statistical analyses, confirm that the following items are present in the figure legend, table legend, main text, or Methods section.

n/a	Confirmed	
☐	☒	The exact sample size (*n*) for each experimental group/condition, given as a discrete number and unit of measurement
☐	☒	A statement on whether measurements were taken from distinct samples or whether the same sample was measured repeatedly
☒	☐	The statistical test(s) used AND whether they are one- or two-sided<br>*Only common tests should be described solely by name; describe more complex techniques in the Methods section.*
☒	☐	A description of all covariates tested
☒	☐	A description of any assumptions or corrections, such as tests of normality and adjustment for multiple comparisons
☐	☒	A full description of the statistical parameters including central tendency (e.g. means) or other basic estimates (e.g. regression coefficient) AND variation (e.g. standard deviation) or associated estimates of uncertainty (e.g. confidence intervals)
☒	☐	For null hypothesis testing, the test statistic (e.g. *F*, *t*, *r*) with confidence intervals, effect sizes, degrees of freedom and *P* value noted<br>*Give P values as exact values whenever suitable.*
☒	☐	For Bayesian analysis, information on the choice of priors and Markov chain Monte Carlo settings
☒	☐	For hierarchical and complex designs, identification of the appropriate level for tests and full reporting of outcomes
☒	☐	Estimates of effect sizes (e.g. Cohen's *d*, Pearson's *r*), indicating how they were calculated

Our web collection on statistics for biologists contains articles on many of the points above.

Software and code

Policy information about availability of computer code

Data collection	Orbitrap Fusion Lumos Tune Application (v3.5.3890), Xcalibur (v4.5.445.18)
Data analysis	MS3 data were searched against a SwissProt human database (SwissProt.2023.4.12; 20,402 entries) using Protein Prospector (v6.3.5; http://prospector.ucsf.edu/prospector/mshome.htm), which was developed by UCSF mass spectrometry facility.<br>Cross-linked peptides were identified through the integration of MS1, MS2 and MS3 data using XL-tools scripts (Wang, et al. Mol Cell Proteomics 2017, 16 (5), 840-854)).<br>Integrative structural modeling was performed using the Python Modeling Interface (PMI) package, a library for modeling macromolecular complexes based the open-source Integrative Modeling Platform (IMP) package version 2.20 (https://github.com/salilab/pmi)

For manuscripts utilizing custom algorithms or software that are central to the research but not yet described in published literature, software must be made available to editors and reviewers. We strongly encourage code deposition in a community repository (e.g. GitHub). See the Nature Portfolio guidelines for submitting code & software for further information.

Data

Policy information about availability of data

All manuscripts must include a data availability statement. This statement should provide the following information, where applicable:

- Accession codes, unique identifiers, or web links for publicly available datasets
- A description of any restrictions on data availability
- For clinical datasets or third party data, please ensure that the statement adheres to our policy

Unless otherwise stated, all data supporting the results of this study can be found in the article, supplementary, and source data files. The mass spectrometry data generated in this study have been deposited to the ProteomeXchange Consortium via the PRIDE partner repository with the dataset identifiers PXD054551 and PXD063825 and will be made publicly available on formal acceptance.

The PXD054551 dataset can be accessed using the following account details: Username: reviewer_pxd054551@ebi.ac.uk ; Password: ZYZElFSqLZnA
The PXD063825 dataset can be accessed using the following account details: Username: reviewer_pxd063822@ebi.ac.uk ; Password: DSTVYd4jmJkx

Research involving human participants, their data, or biological material

Policy information about studies with human participants or human data. See also policy information about sex, gender (identity/presentation), and sexual orientation and race, ethnicity and racism.

Reporting on sex and gender	N/A
Reporting on race, ethnicity, or other socially relevant groupings	N/A
Population characteristics	N/A
Recruitment	N/A
Ethics oversight	N/A

Note that full information on the approval of the study protocol must also be provided in the manuscript.

Field-specific reporting

Please select the one below that is the best fit for your research. If you are not sure, read the appropriate sections before making your selection.

[x] Life sciences [ ] Behavioural & social sciences [ ] Ecological, evolutionary & environmental sciences

For a reference copy of the document with all sections, see nature.com/documents/nr-reporting-summary-flat.pdf

Life sciences study design

All studies must disclose on these points even when the disclosure is negative.

Sample size	There were no population studies here, so no sample size calculation was performed. After initial testing, two biological replicates were chosen for our final experiments to ensure reproducibility.
Data exclusions	No data excluded
Replication	Two biological replicates were performed in-cell cross-linking experiments, three biological replicates were performed for tissue cross-linking experiments.
Randomization	N/A
Blinding	N/A

Reporting for specific materials, systems and methods

We require information from authors about some types of materials, experimental systems and methods used in many studies. Here, indicate whether each material, system or method listed is relevant to your study. If you are not sure if a list item applies to your research, read the appropriate section before selecting a response.

Materials & experimental systems

n/a	Involved in the study
	[x] Antibodies
	[x] Eukaryotic cell lines
[x]	Palaeontology and archaeology
	[x] Animals and other organisms
[x]	Clinical data
[x]	Dual use research of concern
[x]	Plants

Methods

n/a	Involved in the study
[x]	ChIP-seq
[x]	Flow cytometry
[x]	MRI-based neuroimaging

Antibodies

Antibodies used	Streptavidin-HRP (Thermofisher Cat # N100)
Validation	The antibody used was a commercial antibody that has been validated by the company. The western results correlated well with the expected size of the protein. The dilution used was 1:10,000.

Eukaryotic cell lines

Policy information about cell lines and Sex and Gender in Research

Cell line source(s)	HEK293 cell lines stably expressing HTBH-tagged CSN2
Authentication	The cell lines have been auntheticated using affinity purification and western blot analysis.
Mycoplasma contamination	Cells used in this study tested negative for mycoplasma.
Commonly misidentified lines (See ICLAC register)	N/A

Animals and other research organisms

Policy information about studies involving animals; ARRIVE guidelines recommended for reporting animal research, and Sex and Gender in Research

Laboratory animals	Heart tissue was dissected from C57BL/6J/#000664 mice.
Wild animals	N/A
Reporting on sex	N/A
Field-collected samples	N/A
Ethics oversight	Animal experiments were performed according to the protocol approved by the Institutional Animal Care and Use Committee (IACUC) of the University of California, Irvine.

Note that full information on the approval of the study protocol must also be provided in the manuscript.

Plants

Seed stocks	N/A
Novel plant genotypes	N/A
Authentication	N/A
